# Lymphoid malignancies ARNT unstoppable

**DOI:** 10.18632/aging.101010

**Published:** 2016-07-26

**Authors:** Kacie A. Gardella, Richa Tiwary, Casey W. Wright

**Affiliations:** Division of Pharmacology and Toxicology, and The Center for Molecular and Cellular Toxicology, College of Pharmacy, The University of Texas at Austin, Austin, TX 78712, USA

**Keywords:** ARNT, AHR, cancer, autoimmunity

The aryl hydrocarbon receptor nuclear translocator (ARNT; also known as hypoxia inducible factor 1-β) is a member of the basic-helix-loop-helix-Per/ARNT/Sim (bHLH-PAS) family of transcription factors. ARNT is a common binding partner in this family and predominantly heterodimerizes with HIF-1α or the aryl hydrocarbon receptor (AHR), and aids in the recognition of their respective DNA binding sequences [1]. The AHR has been recognized for decades as the mediator of xenobiotic responses to environmental pollutant exposure, which act as ligands for AHR, including polycyclic aromatic hydrocarbons (e.g. benzo(a)pyrene) and halogenated aromatic hydro-carbons such as the high affinity AHR ligand 2,3,7,8-tetrachlorodibenzodioxin [1]. Additionally, AHR signaling plays a role in normal physiology where AHR induction occurs through binding endogenous and natural ligands, most notably tryptophan catabolites [2]. Importantly, physiological AHR signaling is involved in immunomodulation by controlling processes like hematopoiesis, B and T cell differentiation, and immune tolerance [3, 4]. Consequently, interference of normal AHR signaling by environmental toxicants influences the development of autoimmune disorders, including lymphoid malignancies, the incidence of which increases with age. However, little is known about the role of ARNT in AHR immune signaling. Initial studies by our laboratory, and others, into the regulatory role of ARNT in AHR-mediated immunomodulation have suggested that ARNT is an integral cofactor in different immune cell signaling pathways, including NF-κB signaling [5, 6].

ARNT is expressed as two isoforms, isoform 1 and 3, which differ in only 15 amino acids present in isoform 1. Nonetheless, ARNT cellular function had not been considered as the combined activity of the two ARNT isoforms. In our recent Oncotarget priority report it was observed that normal lymphocytes harbor equal levels of isoform 1 and 3, whereas lymphoid malignancies exhibit a shift to higher levels of ARNT isoform 1 to isoform 3 ratios [7]. Through targeted suppression, using isoform specific RNAi to modulate the ARNT isoform ratio, a requirement for ARNT isoform 1 in sustaining proliferation and supporting cell survival was uncovered [7]. We observed that in the absence of ARNT isoform 1, malignant blood cells stopped proliferating due to S-phase cell cycle arrest that is ultimately due to increased p53 stability and activation [7]. Importantly, these phenotypes are specific to suppression of isoform 1, whereas suppression of isoform 3 has no impact on cell proliferation. Subsequently, we have found that suppression of the predominant isoform 1, in various lymphoid cancers, allows basally nuclear AHR to more readily associate with isoform 3, in turn promoting spontaneous basal AHR activity without the requirement for ligand (unpublished observation). Remarkably, this spontaneous basal AHR activity is directly linked to the cell cycle arrest that is observed as a consequence of lowering the ARNT isoform 1 to isoform 3 ratio (unpublished observation). Thus, it appears that the higher protein levels of ARNT isoform 1 present in lymphoid malignancies act as a sink, sequestering basally active AHR away from isoform 3 to suppress AHR signaling and promote cell proliferation (see figure).

**Figure 1:**
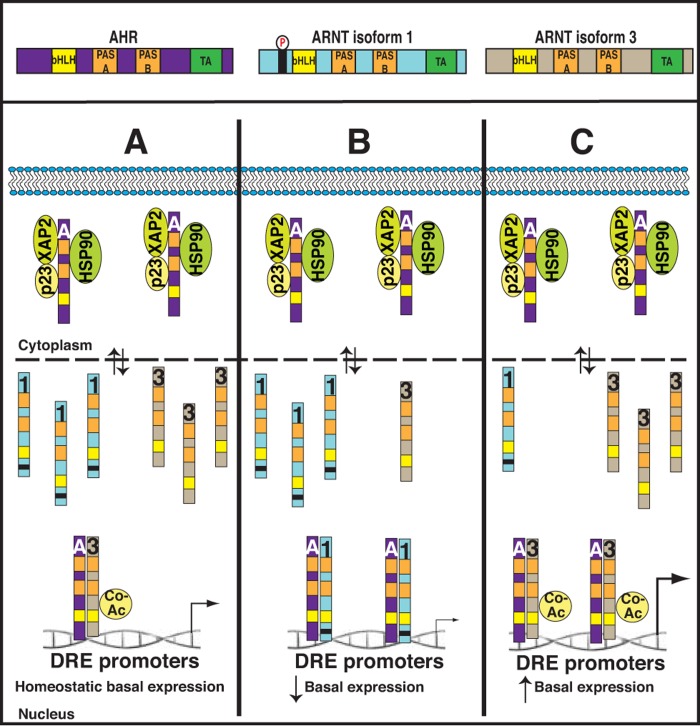
Predictive model for how changes in the ARNT isoform ratio impacts basal AHR activity and cell proliferation Upper Panel: Schematic representation of AHR and the ARNT isoforms. Lower Panel **(A)** Normal lymphocytes exhibit equal levels of ARNT isoform 1 and 3, providing basal AHR activity that keeps the cells at homeostasis. **(B)** Lymphoid malignancies, and possibly activated normal lymphocytes, have high isoform 1:3 ratios that keep basal AHR activity low and allow for cell proliferation. **(C)** Depicts high basal AHR activity after lowering of isoform 1:3 ratios. A=AHR; 1=ARNT isoform 1; 3=ARNT isoform 3; Co-AC=Transcriptional co-activator; DRE=Dioxin response element.

Our work has elucidated a novel regulatory paradigm for AHR signaling by the ARNT isoforms in lymphoid malignancies. As stated, the AHR transcription factor mediates the detrimental effects of environmental contaminants on body tissues and organs [1]. Moreover, AHR has been identified as a crucial factor in controlling physiological immunomodulatory functions [4]. Given the immunomodulatory role of AHR, the goal now is to pharmacologically target AHR for therapeutic intervention of autoimmune disease and cancer. Thus, in order to achieve this goal, much more needs to be accomplished before we clearly understand how the environment impacts the immune system, either beneficially by bolstering our defensive mechanisms or detrimentally by contributing to disease. We predict that characterizing the ARNT isoform functions, in the context of AHR signaling, will provide a prodigious leap forward in this regard, allowing for a significantly improved understanding of AHR biology, and for possible development of ARNT-based interventions for stopping the proliferation of lymphoid malignancies.
